# Identifying Modifiable Risk Factors for Relapse in Patients With Schizophrenia in China

**DOI:** 10.3389/fpsyt.2020.574763

**Published:** 2020-09-11

**Authors:** Wei-Feng Mi, Xiao-Min Chen, Teng-Teng Fan, Serik Tabarak, Jing-Bo Xiao, Yong-Zhi Cao, Xiao-Yu Li, Yan-Ping Bao, Ying Han, Ling-Zhi Li, Ying Shi, Li-Hua Guo, Xiao-Zhi Wang, Yong-Qiao Liu, Zhan-Min Wang, Jing-Xu Chen, Feng-Chun Wu, Wen-Bin Ma, Hua-Fang Li, Wei-Dong Xiao, Fei-Hu Liu, Wen Xie, Hong-Yan Zhang, Lin Lu

**Affiliations:** ^1^Peking University Sixth Hospital, Peking University Institute of Mental Health, NHC Key Laboratory of Mental Health (Peking University), National Clinical Research Center for Mental Disorders (Peking University Sixth Hospital), Beijing, China; ^2^Department of Psychiatry, Affiliated Psychological Hospital of Anhui Medical University, Anhui Mental Health Center, Hefei Fourth People’s Hospital, Hefei, China; ^3^Peking-Tsinghua Center for Life Sciences and PKU-IDG/McGovern Institute for Brain Research, Peking University, Beijing, China; ^4^Key Laboratory of High Confidence Software Technologies (MOE), Department of Computer Science and Technology, Peking University, Beijing, China; ^5^National Institute on Drug Dependence and Beijing Key Laboratory of Drug Dependence, Peking University, Beijing, China; ^6^Department of Pharmacology, School of Basic Medical Sciences, Peking University Health Science Center, Beijing, China; ^7^Department of Psychiatry, The Fourth People’s Hospital of Dalian Jinzhou District, Dalian, China; ^8^Department of Psychiatry, The Sixth People’s Hospital of Hebei Province, Baoding, China; ^9^Department of Psychiatry, Rongjun Hospital of Hebei Province, Baoding, China; ^10^Department of Psychiatry, Beijing HuiLongGuan Hospital, Beijing, China; ^11^Department of Psychiatry, Guangzhou Psychiatric Hospital, Guangzhou, China; ^12^Department of Psychiatry, Jinzhou Kangning Hospital, Jinzhou, China; ^13^Department of Psychiatry, Shanghai Mental Health Center, Shanghai, China; ^14^Department of Psychiatry, The People’s Hospital of Hubei Province, Wuhan, China; ^15^Department of Psychiatry, The Mental Health Center of Xi’an, Xi’an, China

**Keywords:** schizophrenia, relapse, risk factors, predictors, decision-tree model

## Abstract

**Background:**

Preventing relapse of schizophrenic patients is really a challenge. The present study sought to provide more explicit evidence and factors of different grades and weights by a series of step-by-step analysis through *χ^2^* test, logistic regression analysis and decision-tree model. The results of this study may contribute to controlling relapse of schizophrenic patients.

**Methods:**

A total of 1,487 schizophrenia patients were included who were 18–65 years of age and discharged from 10 hospitals in China from January 2009 to August 2009 and from September 2011 to February 2012 with improvements or recovery of treatment effect. We used a questionnaire to collect information about relapse and correlative factors during one year after discharge by medical record collection and telephone interview. The *χ^2^* test and logistic regression analysis were used to identify risk factors and high-risk factors firstly, and then a decision-tree model was used to find predictive factors.

**Results:**

The *χ^2^* test found nine risk factors which were associated with relapse. Logistic regression analysis also showed four high-risk factors further (medication adherence, occupational status, ability of daily living, payment method of medical costs). At last, a decision-tree model revealed four predictors of relapse; it showed that medication adherence was the first grade and the most powerful predictor of relapse (relapse rate for adherence *vs.* nonadherence: 22.9 *vs.* 55.7%, *χ^2^* = 116.36, p < 0.001). The second grade factor was occupational status (employment *vs.* unemployment: 19.7 *vs.* 42.7%, *χ^2^* = 17.72, p < 0.001); the third grade factors were ability of daily living (normal *vs.* difficult: 28.4 *vs.* 54.3%, *χ^2^* = 8.61, p = 0.010) and household income (household income ≥ 3000 RMB *vs.* <3000 RMB: 28.6 *vs.* 42.4%, *χ^2^* = 6.30, p = 0.036). The overall positive predictive value (PPV) of the logistic regression was 0.740, and the decision-tree model was 0.726. Both models were reliable.

**Conclusions:**

For schizophrenic patients discharged from hospital, who had good medication adherence, more higher household income, be employed and normal ability of daily living, would be less likely to relapse. Decision tree provides a new path for doctors to find the schizophrenic inpatient’s relapse risk and give them reasonable treatment suggestions after discharge.

## Introduction

Schizophrenia is a severe psychiatric disorder that has profound effects on both individuals and society. Medical costs and productivity losses that are associated with relapse in schizophrenia are enormous (1, 2). The costs for patients who relapse are approximately three-times higher than patients who do not relapse. According to the American Psychiatric Association (3), the main goal of treatment strategies for the stabilization phase of schizophrenia is to minimize the likelihood of relapse. This emphasizes the importance of identifying and modifying the factors that determine clinical relapse in schizophrenia. Thus, developing effective prevention strategies that may contribute to lowering relapse rates and associated costs is important.

Relapse is a multidimensional event, and prognostic risk factors for relapse in schizophrenia are a longstanding, complex topic. Despite a large body of research that evaluated the factors associated with the relapse of psychosis in schizophrenia, these studies mainly focused on only a few factors, including maintenance medication, substance abuse, family support, and social adjustment (4–11). Additionally, the models that have been used to predict relapse, such as multiple regression or Cox proportional hazard regression, are relatively simplistic. Various factors that are associated with the relapse of different aspects of schizophrenia, such as in first-episode schizophrenia patients, have been investigated, including the number of relapses, reductions of medication, medical costs, and cognitive factors (9, 12–19). However, there is still lack of consensus regarding the predictors of relapse in schizophrenia, which is likely attributable to different periods of observation, statistical methods, and healthcare systems.

In the present study, we conducted a large, multicenter, retrospective, observational investigation of schizophrenia patients in 10 psychiatric hospitals in China. We collected the following information: (1) general demographic information, (2) clinical characteristics, and (3) the living environment and information on social life. We sought to test the consistency of risk factors for relapse in patients with schizophrenia 1 year after hospital discharge and evaluate the interactions between these factors. Our goal was to provide more explicit evidence and factors of different grades and weights that may contribute to controlling relapse of the patients and improving the clinical prognosis.

## Materials and Methods

### Participants

The patients included in the present study were outpatients with schizophrenia screened in 10 psychiatric hospitals in seven provinces in China. Patients were screened by reviewing their medical records. All of the patients were of Han Chinese origin and received treatment with antipsychotic agents after discharge from the hospital. The inclusion criteria were the following: (1) 18–65 years of age, (2) the diagnosis of schizophrenia by eligible hospitals according to the international statistical classification of diseases and related health problems, 10th revision (ICD-10), and (3) discharged from the hospital with recovery or improvement from two parts of time periods: from January 2009 to August 2009 and from September 2011 to February 2012. Patients were excluded if therapeutic effect information were unclear and had residual core symptoms at hospital discharge. The study received approval from the central ethics community of the Ethics Committee of Peking University Sixth Hospital, and it was recognized by all the other participating hospitals. Verbal informed consent was given to all patients or their caregivers in this survey.

### Materials

This study had a retrospective observational design and was supported by “Survey on antipsychotics compliance and relapse of schizophrenia in China (SACRSC)” project. Our previous study reported high rates of relapse and found that poor medication adherence was the main risk factor for relapse, based on χ^2^ analyses of data from schizophrenia patients who were discharged from hospital between September 2011 and February 2012, and the sample size was 992 (20). And before that our work group had already collected a small sample data which contained about 537 schizophrenic patients who were discharged from the hospital with recovery or improvement from January 2009 to August 2009. And in this study, we combine the data of those two parts to enlarge the sample and try to find some more convincing evidence and predictors to support schizophrenia clinical treatment and prevent relapse. So, the study was actually conducted in two time stages: from January 2010 to August 2010 and from September 2012 to February 2013, which were one year after patients’ discharged.

We used cluster random sampling method to collect patients in both two parts of this study (January 2009 to August 2009, September 2011 to February 2012); research hospitals would randomly select one month during that two time period, and all the patients of that randomly selected month who met the inclusion criteria and did not meet the exclusion criteria were included in the study. And the final sample size was 1,487; 42 patients were not included in this study for lack of key information and meeting the exclusion criteria.

**Figure 1:**
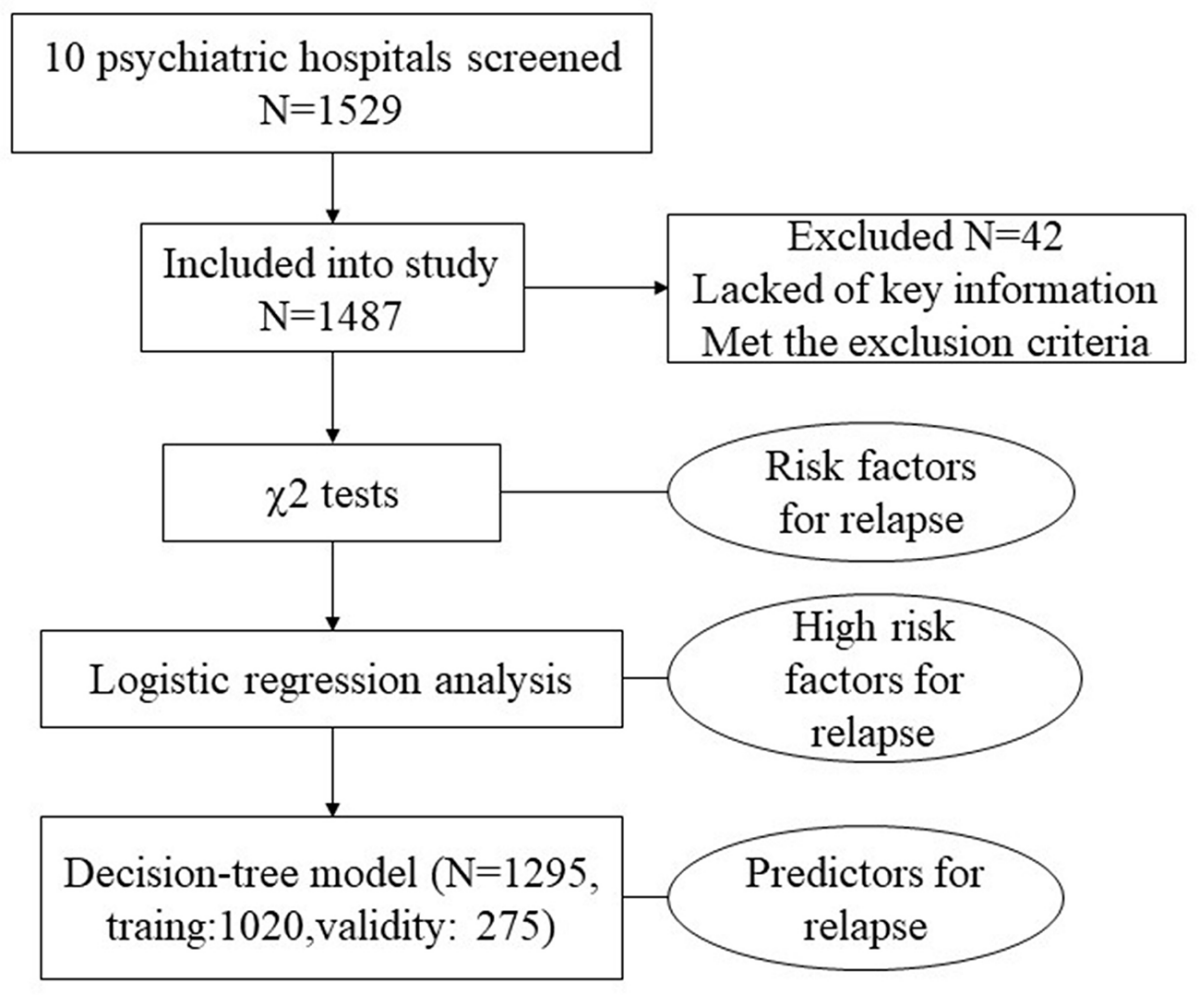
Study design.

We used a questionnaire that was specifically designed for the present survey. The questionnaire included two parts. Part 1 evaluates general demographic information and disease information based on the patients’ medical records of hospital. Disease information included the duration of schizophrenia, age of onset, lifetime number of hospitalizations, duration of last hospitalization, family history of physical and psychiatric illness, and history of smoking/alcohol abuse. Part 2 was designed as a 17-item semi-structured questionnaire to investigate the rate of relapse and rehospitalization and the potential risk factors that might affect relapse. Information on this part was obtained by telephone interview 1 year after hospital discharge. Except for prescribed medications following their doctors’ recommendations, no interventions were performed after hospital discharge during the 1 year prior to the telephone interview. Given the unreliability of self-assessment, patient’s caregivers (*e.g.*, parents, spouses, or siblings) were preferred in the telephone interview. The patients themselves were interviewed only in the case that patients lived alone or patient’s caregivers could not be reached for telephone interview. All patients or their caregivers gave verbal informed consent prior to the telephone interview. If the respondent completed the questionnaire, it represents he/she agreed to participate the survey. And this survey did not include private information. The surveyors of this study were all psychiatrists from the 10 hospitals, and all of them got the same training for the standard operating procedures of each item in the questionnaire to make sure the consistency of the survey. For the study design see **Figure 1**.

#### Definition of Relapse

Currently, no consistent criteria were established in the world to define relapse in schizophrenia. In our study, we defined relapse based on previous study that clinically significant exacerbation of psychotic symptoms during telephone interviews according to self-report or the caregiver’s report. Four operational evaluation criteria were proposed to assess exacerbation of symptoms: a change in antipsychotic treatment, more frequent hospital visits, rehospitalization, and closer supervision because of self-harm, aggressive behavior, and/or suicidal or homicidal ideation (21). Patients who met at least one of the above four criteria were considered to have relapsed (20).

#### Definition of Medication Adherence

In this study, adherence was assessed based on the medication-taking behavior reported by respondents. Discontinuation of drugs was regarded as extreme nonadherence behavior. According to the timing of nonadherence with regard to the prescription or duration of discontinued medication after discharge, adherence was graded as one of three levels: (1) adherent (adherence to medication most of the time, total time of nonadherence less than 2 months, or medication discontinued for less than 2 weeks), (2) moderately nonadherent (adherence to medication half of the time, total time of nonadherence more than 2 months but less than 6 months, or medication discontinued for more than 2 weeks but less than 2 months), and (3) severely nonadherent (almost no adherence to medication, total time of nonadherence more than 6 months, or medication discontinued for more than 2 months) (22, 23).

### Statistical Analysis

After completing the surveys of schizophrenia patients, each hospital sent the questionnaires to the study initiator (Peking University Sixth Hospital) for data analysis. All statistical analysis was performed using Statistiscal Package for the Social Sciences 16.0 software (SPSS, Chicago, IL, USA). We used a series of step-by-step analysis through Chi Square (χ^2^) test, logistic regression analysis, and decision-tree model. The χ^2^ test was performed to identify risk factors associated with relapse. Logistic regression analysis was conducted to model the potentially risk factors of relapse. In logistic regression model, we use medication adherence, occupational status, interpersonal relationships, household income, therapeutic effects, payment of medical costs, family communication, hospital rank as independent variables and relapse as dependent variable (Controlled with gender and medication pattern at time of survey as covariates). Results of the logistic regression models were reported as odds ratios (ORs) with 95% confidence intervals (CIs). No adjustments were made for multiple outcome measures. Collinear analysis between potentially risk factors is reported in **Table S1** in the supplementary material.

A decision tree classification model for relapse was reconstructed using the Chi Square interaction automatic detection analysis (CHIAD) by identifying cutoffs of probabilities for each risk group (24). It gives graded and weighted predictive factors and is helpful for assessing the prognosis of schizophrenia. Specifically, by using the CHIAD method, the most significant risk factor split the sample into two or more subgroups, which are subsequently divided by the next most significant risk factor, and so on until there are no significant risk factors. P-values less than 0.05 were considered to be statistically significant.

#### Decision-Tree Model and Validity

We defined validity as that in a new group of data, those independent variables related to high risk of relapse predicted relapse in schizophrenia by the given model. We take 1,020 of the total samples as training samples to obtain risk factors, and then the remaining 275 of our overall sample are used as verification or validity samples to test the risk factors.

All of the items were analyzed as categorical variables [*e.g.*, medication adherence (nonadherent, adherent)]. More details of the classifications of each factor are provided in **Table 1**. Values of p < 0.05 were considered statistically significant. A 95% confidence interval (CI) of the odds ratio (OR) that overlaps 1 (include OR = 1) indicates no statistical significance. A lower-limit value of the 95% CI that is greater than 1 indicates a greater risk factor or predictor of relapse. An upper-limit value of the 95% CI that is less than 1 indicates a higher likelihood of nonrelapse.

**Table 1-1:** General demographic and disease information of the patients (n=1487).

Items	Frequency/Proportion (*n* [%])	Relapse (*n* [%])
**Sex (male/female)**		
male	659 (44.3%)	214 (37.2%)
female	828 (55.7%)	251 (34.9%)
**Age (years)^a^**		
18–30	389 (26.2%)	100 (29.9%)
31–44	348 (23.4%)	110 (35.5%)
45–65	255 (17.1%)	83 (36.2%)
**Education^b^**		
<9 years	678 (45.6%)	215 (36.3%)
≥9 years	795 (53.5%)	247 (35.7%)
**Occupational status^c^**		
Employed	391 (26.3%)	77 (19.7%)**
Unemployed	887 (59.7%)	379 (42.7%)
**Marital status^d^**		
Married	580 (39.0%)	157 (30.7%)**
Single	715 (48.1%)	233 (37.9%)
Divorced	173 (11.6%)	70 (45.2%)
Widowed	17 (1.1%)	4 (30.8%)
**Cohabitation^e^**		
Live alone	62 (4.2%)	31 (51.7%)*
Living with others	1334 (89.7%)	426 (35.3%)
**Method of payment of medical costs^f^**		
Self-payment	430 (28.9%)	100 (28.3%)**
Medical insurance	1051 (70.7%)	364 (38.9%)
**Residence^g^**		
Urban	771 (51.8%)	248 (36.2%)
Rural	661 (44.5%)	199 (35.6%)
**Family members^h^**		
≤3	612 (41.2%)	227 (37.2%)**
4–8	245 (16.5%)	59 (24.1%)
**Household income^i^**		
≥3,000 RMB	550 (37.0%)	157 (28.6%)**
<3,000 RMB	548 (36.9%)	231 (42.4%)

^a^Four hundred ninety-five patients (33.3%) did not provide this information. ^b^ Fourteen patients (0.9%) did not provide this information. ^c^Two hundred nine patients (14.1%) did not provide this information. ^d^ Two patients (0.1%) did not provide this information. ^e^Ninety-one patients (6.1%) did not provide this information. ^f^Six patients (0.4%) did not provide this information. ^g^Fifty-five patients (3.7%) did not provide this information. ^h^Six hundred thirty patients (42.4%) did not provide this information. ^i^Three hundred eighty-nine patients (26.2%) did not provide this information. *p < 0.05, **p < 0.001.

**Table 1-2:** General demographic and disease information of the patients (*n* = 1487).

Items		Relapse [*n* (%)]
**Disease course (years) (mean ± SD)**	8.85 ± 8.45	
**Disease course [*n* (%)]**		
≤5 years^a^	686 (46.1%)	194 (33.0%)
>5 years	796 (53.5%)	271 (38.3%)
**Age of onset (years) [mean ± SD (range)]^b^**	26.09 ± 9.61 (5–68)	
**Age of onset (*n* [%])**		
<18 years	308 (20.7%)	104 (38.1%)
18–30 years	740 (49.8%)	230 (36.2%)
31–44 years	354 (23.8%)	113 (35.9%)
>45 years	80 (5.4%)	18 (25.0%)
**Number of hospitalizations for schizophrenia [mean ± SD (range)]^c^**	2.94 ± 2.41 (1–20)	
**Number of hospitalizations for schizophrenia [*n* (%)]**		
1	508 (34.2%)	128(29.0%)
2	389 (26.2%)	120 (35.7%)
≥3	584 (39.3%)	217(42.1%)
**Diagnosis (*n* [%])^d^**		
Paranoid schizophrenia	906 (60.9%)	295 (37.0%)
Undifferentiated schizophrenia	376 (25.3%)	122 (37.3%)
Hebephrenic schizophrenia	45 (3.0%)	7 (16.3%)
Catatonic schizophrenia	20 (1.3%)	7 (43.8%)
Residual schizophrenia	11 (0.7%)	4 (44.4%)
Simple schizophrenia	14 (0.9%)	3 (23.1%)
Unknown	90 (6.1%)	20 (27.8%)
**Period of hospitalization 1 year before study (days) [mean ± SD (range)]**	90.75 ± 197.84 (3–2205)	
**Period of hospitalization 1 year before study [*n* (%)]**		
≥2 months	549 (36.9%)	194 (39.4%)
<2 months	938 (63.1%)	271 (33.8%)
**Treatment effect of hospitalization 1 year before study [*n* (%)]**		
Recover	346 (23.3%)	77 (26.2%)**
Improve	1141 (76.7%)	388 (38.8%)
**Family history of mental illness [*n* (%)]^e^**		
Negative	732 (49.3%)	216 (32.9%)
Positive	252 (16.9%)	75 (34.7%)

^a^Five patients (0.3%) did not provide this information. ^b^Five patients (0.3%) did not provide this information. ^c^Six patients (0.4%) did not provide this information. ^d^Twenty-five patients (1.7%) did not provide this information. ^e^Five hundred three patients (33.8%) did not provide this information. **p < 0.001.

**Table 1-3:** General demographic and disease information of the patients (*n* = 1487).

Items	Frequency [*n* (%)]	Relapse [*n* (%)]
**Medication pattern before hospitalization [*n* (%)]^a^**		
According to the doctor's advice	196 (13.2%)	55 (32.2%)*
Irregular medication	206 (13.9%)	71 (39.2%)
Withdrawal	326 (21.9%)	107 (37.0%)
Unknown	66 (4.4%)	16 (27.1%)
Not applicable	195 (13.1%)	44 (25.3%)
**Medication pattern at time of survey [*n* (%)]^b^**		
1 antipsychotic	610 (41.0%)	177 (32.8%)
≥2 antipsychotics	377 (25.4%)	116 (35.2%)
Did not take medication or antipsychotics	5 (0.3%)	0 (0%)
**Antipsychotics patients used when discharged from hospital [*n* (%)]^c^**		
Risperidone	511 (34.4%)	155 (34.8%)
Olanzapine	253 (17.0%)	68 (30.6%)
Clozapine	198 (13.3%)	70 (40.7%)
Quetiapine	155 (10.4%)	55 (42.0%)
Aripiprazole	117 (7.9%)	33 (33.3%)
Other	247 (16.6%)	84 (37.8%)
**Medication side effects^d^**		
No	707 (47.5%)	225 (32.0%)
Yes	405 (27.2%)	135 (33.6%)

^a^Four hundred ninety-eight patients (33.5%) did not provide this information. ^b^Four hundred ninety-five patients (33.3%) did not provide this information. ^c^Six patients (0.4%) did not provide this information. ^d^Three hundred and seventy-five patients (25.2%) did not provide this information. *p < 0.05.

**Table 1-4:** General demographic and disease information of the patients (n=1487).

Items	Frequency [*n* (%)]	Relapse [*n* (%)]
**Medication adherence [*n* (%)]^a^**		
Adherence	786(52.9%)	179(22.9%)**
Nonadherence	511 (34.4%)	284 (55.7%)
**Interpersonal relationships [*n* (%)]^b^**		
Sociable	390 (26.2%)	87 (22.3%)**
Unsociable	481 (32.3%)	205 (42.8%)
**Ability of daily living [*n* (%)]^c^**		
Normal	701 (47.1%)	199 (28.4%)**
Difficult	174 (11.7%)	94 (54.3%)
**Family communication [*n* (%)]^d^**		
Good	667 (44.9%)	212 (31.9%)*
Poor	199 (13.4%)	79 (39.7%)
**Participated in rehabilitation therapy [*n* (%)]^e^**		
No	724 (48.7%)	226 (31.3%)
Yes	36 (2.4%)	10 (27.8%)
**Hospital rank [*n* (%)]**		
Tertiary institution	1114 (74.9%)	315 (33.5%)*
Primary or secondary institution	373 (25.1%)	150 (42.4%)
**Re-hospitalization during last year [*n* (%)]^f^**		
No	1059 (71.2%)	244 (23.1%)**
Yes	222 (14.9%)	218 (98.2%)

^a^One hundred ninety patients (12.8%) did not provide this information. ^b^Six hundred sixteen patients (41.4%) did not provide this information. ^c^Six hundred twelve patients (41.2%) did not provide this information. ^d^Six hundred twenty-one patients (41.8%) did not provide this information. ^e^Six hundred twenty-three patients (48.9%) did not provide this information. fTwo hundred six patients (13.9%) did not provide this information. *p < 0.05, **p < 0.001

## Results

We received a total of 1,529 completed questionnaires, of which 1,487 were eligible for inclusion criteria in the statistical analysis (97.3%). For these patients, the main age was (35.45 ± 11.48) years; of them, 828 were female (55.7%), and 659 were male (44.3%). Information on relapse was obtained from 1,295 of the patients, of which 465 (35.9%) relapsed. We defined “moderately nonadherent” and “severely nonadherent” cases together as nonadherent, and the proportion of “adherent” participants was 52.9% (786/1,487). The general demographic information and general disease information of the participants are presented in **Table 1**, and we found the patients’ social and family support factors may greatly influence their relapse.

### Risk Factors for Relapse

χ^2^ tests were conducted to determine the risk factors for relapse. Highly related risk factors are presented in **Table 2**, and unrelated factors are presented in **Supplemental Table S1**. Multivariable analyses showed that these factors were independent of each other, and no correlation existed between them. Nine risk factors for relapse were identified: (1) In terms of medication adherence, patient who has medication adherence had a lower risk of relapse than those with nonadherence (relapse rate for adherence *vs.* nonadherence: 22.9 *vs.* 55.7%, OR = 4.23, 95% CI = 3.32–5.38). (2) In terms of occupational status, those who were employed had a lower risk of relapse than those unemployed (relapse rate for employment *vs.* unemployment: 19.7 *vs.* 42.7%, OR = 3.04, 95% CI = 2.29–4.04). (3) In terms of interpersonal relationships, patient who has sociable relationships had a lower risk of relapse than that with unsociable relationships (relapse rate for sociability *vs.* unsociability: 22.3 *vs.* 42.8%, OR = 2.61, 95% CI = 1.93–3.52). (4) In terms of ability of daily living, patients with difficult daily living was prone to relapse than those with normal daily living (relapse rate for normal daily living *vs.* difficult daily living: 28.4 *vs.* 54.3%, OR = 3.00, 95% CI = 2.13–4.21). (5) In terms of household income, patients with house income over 3,000 RMB every month has a lower risk of relapse than patients whose house income were lower than 3,000 RMB (relapse rate for household income over 3,000 RMB *vs.* less than 3,000 RMB: 28.6 *vs.* 42.4%, OR = 1.83, 95% CI = 1.43–2.36). (6) In terms of therapeutic effects on discharge, patients who were improved has a higher risk of relapse than those recovered (relapse rate for recovery *vs.* improvement: 26.2 *vs.* 38.8%, OR = 1.78, 95% CI = 1.34–2.38). (7) In terms of method of payment of medical costs, people who self-paid has a lower risk of relapse than those paid *via* medical insurance (relapse rate for self-payment *vs.* medical insurance: 28.3 *vs.* 38.9%, OR = 0.62, 95% CI = 0.48–0.81). (8) In terms of family communication, patients with good communication between family members has a lower risk of relapse than those with poor communication (relapse rate for good communication *vs.* poor communication: 31.9 *vs.* 39.7%, OR = 1.49, 95% CI = 1.02–2.17), and (9) in terms of hospital rank, people who went to tertiary institution verse primary or secondary institution for treatment had a lower risk of relapse (relapse rate for tertiary institution *vs.* primary or secondary institution: 33.5 *vs.* 42.4%, OR = 1.46, 95% CI = 1.14–1.89; **Table 2**).

**Table 2:** Risk factors associated with relapse.

Variables		Relapse				
		rate (*n* [%])	*χ^2^*	*p*	OR	95%CI
Medication adherence^a^	Adherence	179(22.9%)	144.01	<0.001**	4.23	3.32–5.38
	Nonadherence	284 (55.7%)				
Occupation status^b^	Employed	77 (19.7%)	66.41	<0.001**	3.04	2.29–4.04
	Unemployed	379 (42.7%)				
Interpersonal relationships^c^	Sociable	87 (22.3%)	41.40	<0.001**	2.61	1.93–3.52
	Unsociable	205 (42.8%)				
Ability of daily living^d^	Normal	199 (28.4%)	39.84	<0.001**	3.00	2.13–4.21
	Difficult	94 (54.3%)				
Household income^e^	≥ 3000 RMB	157 (28.6%)	22.62	<0.001**	1.83	1.43–2.36
	< 3000 RMB	231 (42.4%)				
Therapeutic effects 1 year before study^f^	Recover	77 (26.2%)	15.60	<0.001**	1.78	1.34–2.38
	Improve	388 (38.8%)				
Payment of medical costs^g^	Self-payment	100 (28.3%)	12.41	<0.001**	0.62	0.48–0.81
	Medical insurance	364 (38.9%)				
Family communication^h^	Good	212 (31.9%)	4.32	0.038*	1.49	1.02–2.17
	Poor	79 (39.7%)				
Hospital rank^i^	Tertiary institution	315 (33.5%)	8.80	0.003*	1.46	1.14–1.89
	Primary or secondary institution	150 (42.4%)				

^a^Medication adherence (0 = nonadherent; 1 = adherent), ^b^Occupational status (0 = employed; 1 = unemployed). ^c^Interpersonal relationships (0 = sociable; 1 = unsociable). ^d^Ability of daily living (0 = normal; 1 = difficult). ^e^Household income (0 = household income ≥ 3,000 RMB; 1 = household income < 3,000 RMB). ^f^Therapeutic effects (0 = recover; 1 = improve). ^g^Payment of medical costs (0 = medical insurance; 1 = self-payment). ^h^Family communication (0 = good; 1 = poor). ^i^Hospital rank (0 = tertiary institution; 1 = primary or secondary institution). *p < 0.05, **p < 0.001.

In χ^2^ tests, we also found statistical significance in different groups’ relapse rate of marital status, cohabitation, family members, and medication pattern before hospitalization, re-hospitalization during the last year (**Table 1**). But after correction, all the above five variables had too large difference in group sample number (for example: in a variable, one group number was more than 1,300 and the other was less than 100), and the groups in the same variable cannot combine at all, and some variables had significant correlation (cohabitation and family members). Thus, the statistical tests were not convincing and stable enough for those five variables, and we did not include them in our results and next step analysis. We also showed nonsignificant variables for relapse of χ^2^ tests in **Supplemental Table S2**.

### High-Risk Factors for Relapse

Logistic regression analysis was conducted to identify the stronger risk factors of relapse. We found that medication adherence (OR **= 4**.07, 95% CI = 2.94–5.64, p < 0.001), occupational status (OR = 2.50, 95% CI = 1.65–3.79, p < 0.001), ability of daily living (OR = 1.88, 95% CI = 1.27–2.77, p = 0.002), and method of payment of medical costs (OR = 0.56, CI = 0.37–0.84, p = 0.005) highly predicted relapse (**Table 3**). For schizophrenic patient discharged from the hospital, those who had good medication adherence, employed, normal ability of daily living and self-payment, would less likely to relapse.

**Table 3:** Logistic regression analysis of significant risk factors of relapse.

Variables		Relapse		
		*p*	OR	95% CI
Medication adherence^a^	Adherent *vs.* nonadherent	<0.001**	4.07	2.94–5.64
Occupational status^b^	Employed *vs.* unemployed	<0.001**	2.50	1.65–3.79
Ability of daily living^c^	Normal *vs.* difficult	0.002*	1.88	1.27–2.77
Payment of medical costs^d^	Medical insurance *vs.* self-payment	0.005*	0.56	0.37–0.84

^a^Medication adherence (0 = nonadherent; 1 = adherent). ^b^Occupational status (0 = unemployed; 1 = employed). ^c^Ability of daily living (0 = difficult; 1 = normal). ^d^Payment of medical costs (0 = self-payment; 1 = medical insurance). *p < 0.05, **p < 0.001.

### Predictors for Relapse

**Figure 2:**
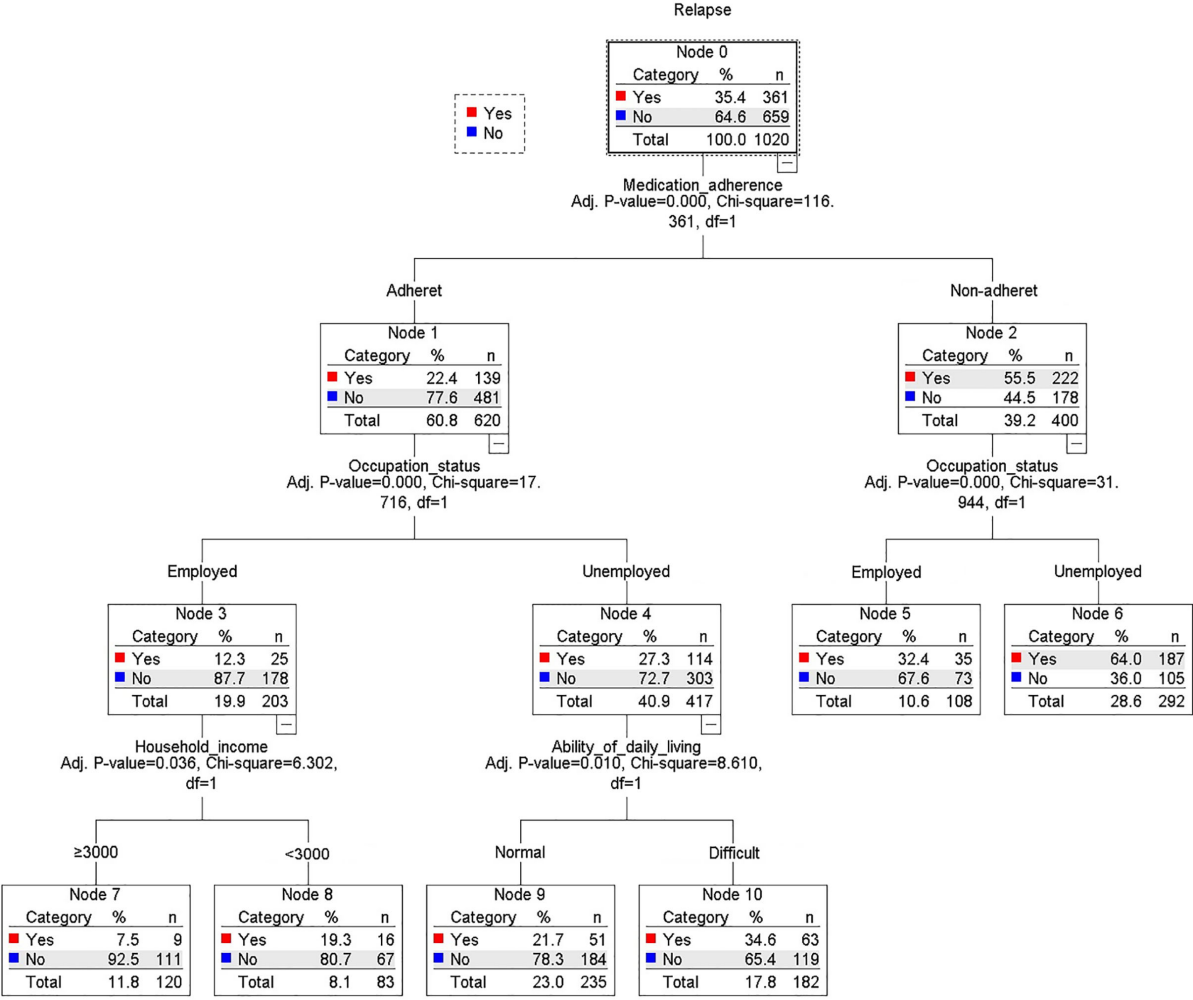
Decision-tree model of predictors of relapse (training model, n = 1,020).

As expected, poor medication adherence, unemployment, poor ability of daily living, and low household income highly predicted relapse (training samples see **Figure 2**, validity samples see **Supplementary Materials**, **Figure S1**). Medication adherence was the first differentiator (or grade, node) of cases (**Figure 2**). Occupational status produced the second split (it was significant in both medication adherent and nonadherent group; adherent: p < 0.001, nonadherent: p < 0.001). Among the patients who were adherent and employed, the third predictor of relapse was lower household income (<3,000 RMB). Among the patients who were adherent and unemployed, the third-grade predictor of relapse was difficult ability of daily living.

The predicted performance of the logistic regression analysis and decision tree model is presented in **Table 4**. The decision-tree model had a positive predictive value (PPV) of 0.726, meaning that it correctly predicted 72.6% of relapse cases. The overall PPV of the logistic regression was 0.740. Both models reliably predicted relapse.

**Table 4:** Predictive performance of logistic regression analysis and decision-tree model.

	Observed^a^	Predicted		
		No (*n*)	Yes (*n*)	% Correct
Logistic regression	No	455	76	85.7
	Yes	133	139	51.1
	Overall %			74.0
Decision-tree training	No	554	105	84.1
	Yes	174	187	51.8
	Overall %	71.4	28.6	72.6
Decision-tree Test	No	142	29	83.0
	Yes	49	55	52.9
	Overall %	69.5	30.5	71.6

^a^“Observed” indicates actual observation of relapse or nonrelapse. “Predicted” indicates precomputed prevalence of relapse or nonrelapse. “No” indicates nonrelapse. “Yes” indicates relapse.

## Discussion

In the present study, we found that poor medication adherence, unemployment, difficult ability of daily living, medical insurance of medical costs, and low household income were highly related to relapse by using χ2 tests, logistic regression analysis, and decision-tree models. As expected, poor medication adherence was the most significant predictor of relapse, and 55.8% of the nonadherent patients relapsed compared with only 23% of the adherent patients (**Figure 2**).

Medication nonadherence is a critical factor of relapse in schizophrenia patients. Poor adherence increases the risk of relapse and hospital readmission (17, 19, 25–27). The consequences of inadequate medication adherence include poor clinical outcomes, increases in morbidity and disability, a lower quality of life, decreases in work and personal productivity, and an increase in healthcare costs (14, 28). The causes of nonadherence are multifactorial. Negative attitudes about medications play an important role in nonadherence (29, 30). Medication adherence is one of the most effective means of preventing relapse in schizophrenia (31, 32).

Unemployment is a high-risk factor for relapse in schizophrenia patients. Reports of the prevalence of unemployment showed that 75–90% of adults with schizophrenia are unemployed, and unemployment contributes to disease recurrence (33). Theoretically, unemployment may associate with another high-risk factor, for example, household income. However, according to colinear analysis, there was no collinearity between these two factors. One possible explanation for this is that the household income did not correctly reflect patients own income, and only small part (4.2%) of the patients were living alone. We also found that difficult ability of daily living predicted relapse in schizophrenia patients 1 year after hospital discharge. Social skills’ training is highly recommended to improve schizophrenia patients’ deficits in the ability of daily life (34). In addition to many well-documented factors that are associated with psychotic relapse, our work also emphasizes the important role of self-payment of medical costs and household income in relapse. An explanation offered by the decision tree model was that patients with lower income or unemployed appeared as predictors of medical costs and household income. These two risk factors were not regarded as predictors of relapse in previous studies that were conducted in developed countries (23, 25, 35, 36), which may be attributable to these countries’ comparatively advanced healthcare systems and public services. Hospitals in China are organized according to a three-tier system (*i.e.*, primary, secondary, and tertiary institutions) that recognizes a hospital’s ability to provide medical care (29). Patients who receive treatment in nontertiary institutions (primary and secondary institutions) are more likely to relapse; that was also an important result of this study, and in the future, we may do more work to improve primary and secondary institutions’ mental disease treatment.

In the present study both logistic regression and decision-tree models were used to identify high-risk factors and predictors for relapse. Since we use categorical variables and some variables may have interacted with each other, while, logistic regression cannot properly deal with problems of nonlinear and interactive effects of explanatory variables (37). Therefore, it is necessary to verify the findings of logistic regression by decision trees. In addition, the decision tree model has some advantages over generalized linear models (logistic regression). First, compared with generalized linear models, decision tree model is easier to understand because the results exported in the decision tree model were resembling clinical decision-making processes. Second, tree-structures are more flexible to distribute the response variable without pre-assumption. And we found an article that used the same strategy with our study in which they used χ^2^ followed by logistic regression and decision tree consecutively (38). Our results from the decision-tree analysis were corroborated by both the logistic regression and χ^2^ analyses. Moreover, we verified the results using data from 275 separate patients (approximately 20% of the total number of patients who were included in the study). Therefore, the results of these models were robust, and our findings may provide further evidence for preventing relapse of schizophrenia patients.

It is also important to mention the factors that did not significantly predict first year relapse in patients with schizophrenia. In the present study, the initial χ^2^ tests indicated that smoking, alcohol dependence, rural residence, side effects of medication, a low level of education (<9 years), period of hospitalization (≤2 months), disease course (>5 years), and a family history of schizophrenia were not significantly related to relapse at the end of the first year. These findings contrast with previous studies (9, 39, 40). This discrepancy might be attributable to differences in the definition of relapse or periods of observation. Further examinations of these factors in different situations are needed to provide more precise recommendations for the clinical prognosis of relapse.

The present study has some limitations. First, the study was not designed to assess adherence to specific antipsychotic medications; we only controlled medication pattern as covariate in both regression and decision tree models. We focused on external risk factors beyond pharmacological effects that may indirectly affect medication adherence and relapse. Second, the data may have been influenced by a “social desirability response” during the telephone interview, which refers to the tendency of survey respondents to answer questions in a manner that will be viewed favorably by others (41). Cultural idioms provide some protection against stigma among respondents in China (42, 43). Third, although all of the investigators were trained and licensed psychiatrists and information on general demographic information and disease information was obtained from the patients’ medical records of hospital, data about relapse were provided by the respondents which may give false information, and it was easily prone to recall bias. It is an inevitable limitation for retrospective study. Fourth, risk factors associated with relapse in schizophrenia did not imply casual relationships. Further study was needed to explore the predictor of relapse in patients. However, it may not often feasible to study the relationships because of various confounding variables in the real world.

## Conclusions

In summary, this study considered several important variables and used three statistical models to evaluate the risk factors, high-risk factors or predictors associated with relapse in schizophrenia. Our work provides strong evidence that poor medication adherence, unemployment, difficult ability of daily living, medical insurance of medical costs, and low household income were main predictors of relapse in schizophrenia patients 1 year after hospital discharge. Medication adherence was the first-grade predictors of relapse. Clinicians should be fully aware of this risk and provide accurate information to patients about the risk of relapse. Further studies evaluating intervention strategies focused on reducing relapse and improving insight into schizophrenia should be encouraged.

## Data Availability Statement

The raw data supporting the conclusions of this article will be made available by the authors, without undue reservation.

## Ethics Statement

The studies involving human participants were reviewed and approved by the Ethics Committee of Peking University Sixth Hospital and recognized by all the other participating hospitals. The patients/participants provided their written informed consent to participate in this study.

## Author Contributions

W-FM conceived of the study. W-FM, and T-TF participated in its design and coordination and drafted the manuscript. X-MC and J-BX performed the statistical analysis and interpretation of the data. W-FM, ST, T-TF, X-MC, Y-PB, YH, L-ZL, YS, L-HG, X-ZW, Y-QL, Z-MW, J-XC, F-CW, W-BM, H-FL, W-DX, and F-HL participated in the coordination of the study and performed the measurements. WX, H-YZ, and LL contributed to the critical revision of the manuscript. All authors contributed to the article and approved the submitted version.

## Funding

This work was supported by the Beijing Municipal Science and Technology Commission (project number: Z151100003915121, Z181100001718157), National Key Research and Development Program of China (project number: 2016YFC1304400), National Science and Technology Major Project for IND (project number: 2018ZX09734-005). The funding organization had no influence on the design and conduct of the study, collection, management, analysis, and interpretation of the data, preparation, review, or approval of the manuscript, or the decision to submit the manuscript for publication.

## Conflict of Interest

The authors declare that the research was conducted in the absence of any commercial or financial relationships that could be construed as a potential conflict of interest.

The handling editor declared a shared affiliation with one of the authors [H-FL] at time of review.

## Abbreviations

CHIAD, Chi Square interaction automatic detection analysis; CI, confidence interval; OR, odds ratio; PPV, positive predictive value.
