# Supplementary material for: Identifying Modifiable Risk Factors for Relapse in Patients With Schizophrenia in China
Source: Front Psychiatry. 2020 Sep 11;11:574763. doi: 10.3389/fpsyt.2020.574763 (PMC7518216; doi:10.3389/fpsyt.2020.574763)

**Supplementary Materials**

**Methods**

**The following 10 hospitals participated in screening patients: Peking University Sixth Hospital (Beijing, China), The Fourth People’s Hospital of Dalian Jinzhou District (Dalian, China), The Sixth People’s Hospital of Hebei Province (Baoding, China), Rongjun Hospital of Hebei Province (Baoding, China), Beijing HuiLongGuan Hospital (Beijing, China), Guangzhou Psychiatric Hospital (Guangzhou, China), Jinzhou Kangning Hospital (Jinzhou, China), Shanghai Mental Health Center (Shanghai, China), The People’s Hospital of Hubei Province (Wuhan, China), and The Mental Health Center of Xi’an (Xi’an, China).**

**Table S1. Variance Inflation Factors (VIF) for covariates used in logistic regression model and decision-tree model.**

|  | **VIF** |
| --- | --- |
| **Medication adherence** | **1.09** |
| **Occupation status** | **1.27** |
| **Interpersonal relationship** | **1.42** |
| **Ability of daily living** | **1.35** |
| **Household income** | **1.15** |
| **Therapeutic effects 1 year before study** | **1.06** |
| **Payment of medical costs** | **1.06** |
| **Family communication** | **1.04** |
| **Hospital rank** | **1.11** |
| **Medication pattern when discharged from hospital** | **1.04** |
| **Sex** | **1.04** |

**Table S2. Nonsignificant factors for relapse.**

| **Variables** | | **Frequency**  **(*n* [%])** | **Relapse** | | | | |
| --- | --- | --- | --- | --- | --- | --- | --- |
|  |  |  | **rate (*n* [%])** | ***χ^2^*** | ***p*** | **OR** | **95%CI** |
| **Smoking^a^** | **No** | **677 (45.5%)** | **219 (32.5%)** | 2.28 | 0.131 | 1.29 | 0.93-1.81 |
|  | **Yes** | **202 (13.5%)** | **73 (38.6%)** |  |  |  |  |
| **Alcohol^b^** | **No** | **774 (52.1%)** | **275 (33.8%)** | 0.22 | 0.640 | 0.86 | 0.45-1.63 |
|  | **Yes** | **66 (4.4%)** | **14 (30.4%)** |  |  |  |  |
| **Residence^c^** | Urban | 771 (51.8%) | 248 (36.2%) | 0.05 | 0.825 | 0.97 | 0.77-1.23 |
|  | Rural | 661 (44.5%) | 199 (35.6%) |  |  |  |  |
| **Medication side effects^d^** | **No** | **707 (47.5%)** | 225 (32.0%) | 0.31 | 0.580 | 1.08 | 0.83-1.40 |
|  | **Yes** | **405 (27.2%)** | **135 (33.6%)** |  |  |  |  |
| **Education level^e^** | < 9 years | 678 (45.6%) | 215 (36.3%) | 0.05 | 0.816 | 1.03 | 0.82-1.29 |
|  | ≥ 9 years | 795 (53.5%) | 247 (35.7%) |  |  |  |  |
| **Period of hospitalization 1 year before study^f^** | ≥ 2 months | **549 (36.9%)** | 194 (39.4%) | 3.12 | 0.077 | 1.24 | 0.98-1.56 |
|  | < 2 months | **938 (63.1%)** | **271 (33.8%)** |  |  |  |  |
| **Disease course^g^** | ≤ 5 years | 686 (46.1%) | 194 (33.0%) | 3.81 | 0.051 | 1.26 | 1.00-1.58 |
|  | > 5 years | 796 (53.5%) | 271 (38.3%) |  |  |  |  |
| **Family history of** [**schizophrenia**](javascript:void(0);)**^h^** | Negative | 732 (49.3%) | 216 (32.9%) | 0.24 | 0.627 | 1.08 | 0.78-1.50 |
|  | Positive | 252 (16.9%) | 75 (34.7%) |  |  |  |  |

**Note:** ^a^Smoking (0 = no; 1 = yes), **five hundred and fifty-two patients (37.1%) did not provide this information.** ^b^Alcohol (0 = no; 1 = yes), **five hundred and ninety patients (39.7%) did not provide this information.** ^c^Residence (0 = rural; 1 = urban), **fifty-five patients (3.7%) did not provide this information**. ^d^Medication side effects (0 = no; 1 = yes), **three hundred and seventy-five patients (25.2%) did not provide this information**. ^e^Education level (0 = ≥ 9 years; 1 = < 9 years), **Fourteen patients (0.9%) did not provide this information**. ^f^Period of hospitalization (0 = ≥ 2 months; 1 = < 2 months). ^g^Disease course (0 = ≤ 5 years; 1 = > 5 years), **five patients (0.3%) did not provide this information**. ^h^Family history of [schizophrenia](javascript:void(0);) (0 = no; 1 = yes), **five hundred three patients (33.8%) did not provide this information**.

**Figure S1. Decision-tree model for predictors of relapse (verification model, n = 275).** Medication adherence (0 = non-adherent; 1 = adherent), occupational status (0 =unemployed; 1 = employed), household income (0 =< 3000 RMB; 1 =≥ 3000 RMB), ability of daily living (0 = difficult; 1 = normal).


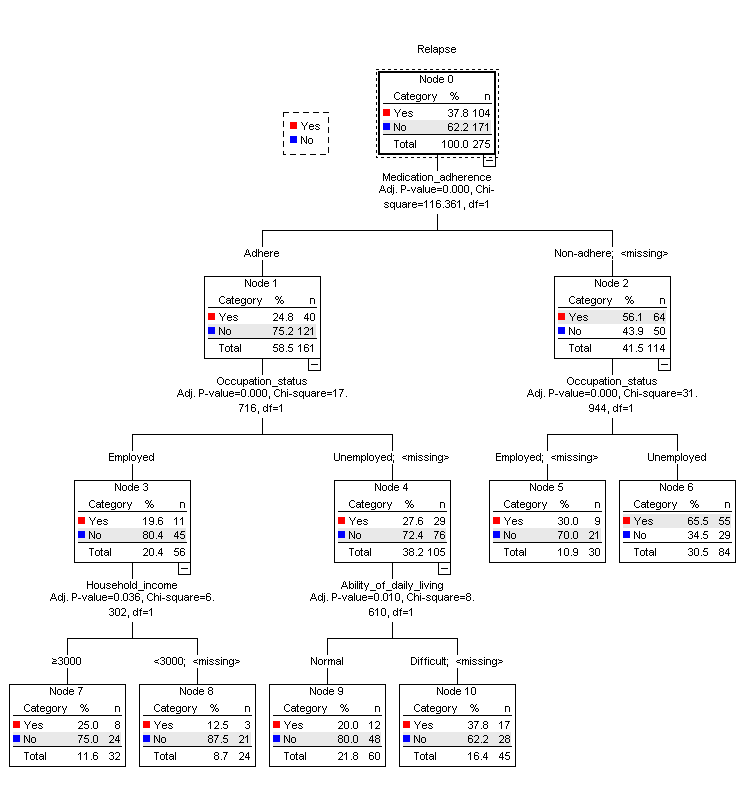

Supplement: Supplementary file 1 [file DataSheet_1.docx]
